# Snake River alfalfa virus, a persistent virus infecting alfalfa (*Medicago sativa* L.) in Washington State, USA

**DOI:** 10.1186/s12985-023-01991-7

**Published:** 2023-02-19

**Authors:** Olga A. Postnikova, Brian M. Irish, Jonathan Eisenback, Lev G. Nemchinov

**Affiliations:** 1 Molecular Plant Pathology Laboratory, USDA/ARS, Beltsville Agricultural Research Center, Beltsville, MD USA; 2 School of Plant and Environmental Sciences, Virginia Tech, Blacksburg, VA USA; 3 USDA/ARS Plant Germplasm Introduction Testing and Research Unit, Prosser, WA USA

**Keywords:** Alfalfa, *Medicago sativa* L., Snake River alfalfa virus, Endornavirus, Seed transmission

## Abstract

Here we report an occurrence of Snake River alfalfa virus (SRAV) in Washington state, USA. SRAV was recently identified in alfalfa (*Medicago sativa* L.) plants and western flower thrips in south-central Idaho and proposed to be a first flavi-like virus identified in a plant host. We argue that the SRAV, based on its prevalence in alfalfa plants, readily detectable dsRNA, genome structure, presence in alfalfa seeds, and seed-mediated transmission is a persistent new virus distantly resembling members of the family *Endornaviridae*.

**Supplementary Information:**

The online version contains supplementary material available at 10.1186/s12985-023-01991-7.

## Main text

Snake River alfalfa virus (SRAV) was recently identified from alfalfa plants and thrips *Frankliniella occidentalis* collected in the Minidoka and Twin Falls counties of Idaho, USA [1]. Based on the genome structure and phylogeny of its RNA-dependent RNA polymerase (RdRp), the authors hypothesized that SRAV is the first flavi-like virus identified in a plant host [1]. The SRAV polyprotein, however, contained no predicted helicase domain found in all flaviviruses.

**Fig. 1:**
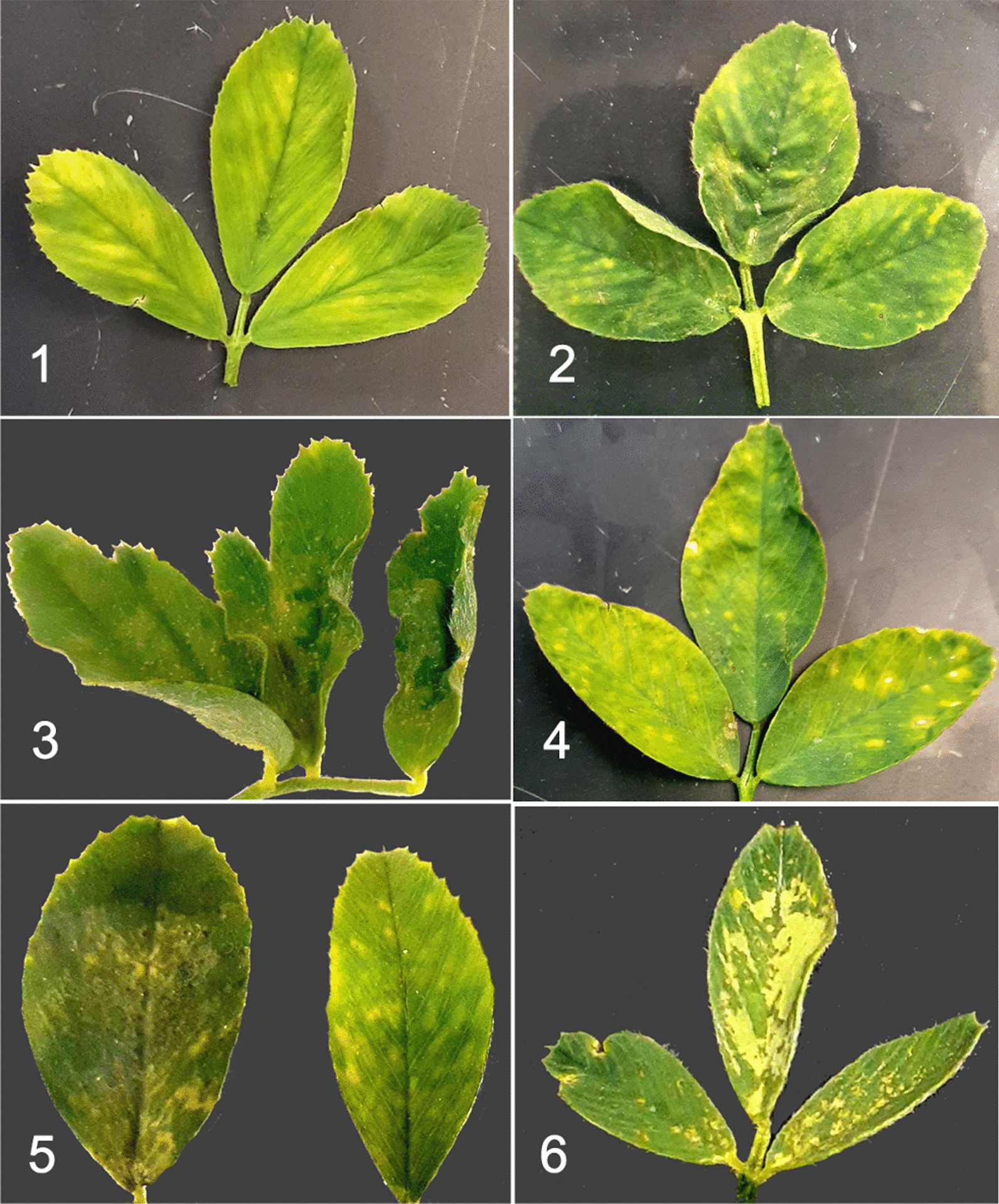
Examples of different symptomatology observed on the leaves of alfalfa (*Medicago sativa* L) plants infected with Snake River alfalfa virus (SRAV-WA1) in Washington State, USA. Sample 1 contained 7,016 reads of SRAV and 11,797 covered bases; sample 2 contained 10,684 reads mapped to the virus and 11,699 covered bases; sample 3 contained 47,175 reads mapped to the virus and 11,838 covered bases; sample 4 contained 71,267 viral reads and 11,811 covered bases; sample 5 contained 8,079 reads and 11,872 covered bases (100% vs. reference genome); and sample 6 contained 19,539 viral reads and covered 11,795 bases. Multiple viral, fungal and bacterial pathogens were also identified in samples in which SRAV was found. These included alfalfa mosaic virus, bean leaf roll virus, pea streak virus, partitiviruses, amalgavirus, lucerne transient streak virus, *Alternaria alternata*, *Alternaria arborescens, Bipolaris* spp., *Stemphylium lycopersici*, *Fusarium* spp., *Pseudomonas* spp., *Erwinia* spp., etc. thus emphasizing the importance of pathobiome in signs and symptoms of disease

To date, no occurrences of SRAV have been reported in alfalfa or on different hosts from other locations. In this work, applying high-throughput sequencing (HTS), we detected SRAV in 50 individual alfalfa plant samples collected from 10 commercial fields in Grant County, WA. Plants used for RNA extraction displayed a diverse symptomatology that occasionally correlated with the symptoms allegedly reported for SRAV, such as yellowing and vein clearing (Fig. 1), [1]. These symptoms, however, were likely due to the presence of multiple co-infecting pathogens in the same plants.

Total RNA was extracted using Promega Maxwell® RSC Plant RNA Kit (Promega Corp., Fitchburg, WI). Library preparation was performed with Illumina TruSeq Stranded Total RNA with Ribo-Zero kit (Illumina Inc., San Diego, CA), and the sequencing platform used was HiseqX10 (PE150) (Omega Biosciences, Norcross, GA). Bioinformatic pipeline included adapter trimming followed by de-novo assembly of reads, unmapped to *M. sativa* genome and *M. truncatula* mitochondrion genomes using SPAdes [7] in HMM-guided mode. Phylogenetic analysis was performed with MEGA software [6] using Maximum Likelihood method and bootstrap analysis of 1000 replicates. Conserved RdRp domains for multiple alignment were obtained using InterPro tool (https://www.ebi.ac.uk/interpro/).

All 50 unique alfalfa plant samples contained viral reads, varying in quantity from 46 to 71,267 (Table 1). Total number of reads mapping to the SRAV genome was 1,017,715 with an approximate length of each read 150 nt (Table 1). Several contigs apparently representing the complete genome of the virus were assembled de novo. The sequences varied slightly in length and contained small number of SNPs, indicating a presence of different genetic variants of the virus. Nevertheless, the sequence identity when compared to the reference genome (ON669064) in all cases remained > 99%.

**Table 1:** Occurrence of SRAV-related reads in 10 commercial alfalfa fields (five samples per field) of Grant County, Washington State

Sample ID	Length	Covered %	Covered bases	Total viral reads	Reads/sample	Yield (Mb)	% ≥ Q30
A2-1	11,872	100	11,872	8079	31,990,204	4831	94.56
A1-4	11,872	99.9832	11,870	27,925	37,707,294	5694	94.54
A4-3	11,872	99.9663	11,868	22,147	39,187,338	5917	94.75
B8-4	11,872	99.9579	11,867	15,093	45,330,070	6845	95.26
A3-4	11,872	99.9326	11,864	44,605	44,468,798	6715	94.77
A3-5	11,872	99.7473	11,842	32,167	34,259,318	5173	94.76
B8-2	11,872	99.7136	11,838	47,175	44,581,834	6732	95.21
A5-5	11,872	99.6715	11,833	21,219	45,938,534	6937	94.76
B6-5	11,872	99.6631	11,832	13,815	44,882,040	6777	95.29
A5-4	11,872	99.6462	11,830	8513	34,368,240	5190	94.66
B8-5	11,872	99.621	11,827	53,101	37,624,098	5681	95
A1-2	11,872	99.5788	11,822	28,724	42,968,950	6488	94.74
A3-1	11,872	99.5115	11,814	33,926	42,255,734	6381	94.78
A2-2	11,872	99.4862	11,811	71,267	37,337,248	5638	94.71
B6-1	11,872	99.4862	11,811	17,265	47,716,504	7205	93.95
B7-5	11,872	99.4525	11,807	25,870	31,743,182	4793	95.13
A1-5	11,872	99.4441	11,806	20,408	46,898,746	7082	94.9
B6-4	11,872	99.4104	11,802	24,605	36,660,924	5536	95.13
B7-4	11,872	99.402	11,801	29,217	39,153,470	5912	95.18
B8-1	11,872	99.402	11,801	44,383	33,674,988	5085	94.86
A1-3	11,872	99.3851	11,799	14,704	34,965,248	5280	94.65
A2-5	11,872	99.3851	11,799	27,777	50,933,376	7691	94.69
A4-4	11,872	99.3851	11,799	20,253	31,710,774	4788	94.62
A5-2	11,872	99.3851	11,799	14,048	42,018,256	6345	94.7
B10-4	11,872	99.3851	11,799	16,305	35,022,936	5288	95.1
B8-3	11,872	99.3851	11,799	37,761	36,163,008	5461	95.34
B9-3	11,872	99.3851	11,799	26,815	41,588,106	6280	95.12
B6-3	11,872	99.3767	11,798	30,672	43,616,596	6586	95.3
B9-4	11,872	99.3767	11,798	10,475	50,653,058	7649	95.3
A2-3	11,872	99.3683	11,797	64,184	39,012,034	5891	94.7
A4-1	11,872	99.3683	11,797	12,057	35,547,054	5368	94.6
A5-3	11,872	99.3683	11,797	7016	50,538,150	7631	94.98
B6-2	11,872	99.3683	11,797	8332	55,187,384	8333	95.25
B7-1	11,872	99.3683	11,797	39,608	35,432,658	5350	95.2
B7-3	11,872	99.3683	11,797	28,372	45,878,018	6928	95.27
B9-2	11,872	99.3683	11,797	12,085	43,347,806	6546	95.11
A2-4	11,872	99.3514	11,795	19,539	32,282,770	4875	94.76
A4-5	11,872	99.343	11,794	4425	32,902,614	4968	94.63
B7-2	11,872	99.343	11,794	10,734	35,619,196	5378	95.05
B10-5	11,872	98.8376	11,734	9244	35,500,288	5361	95.15
B9-5	11,872	98.5428	11,699	10,684	37,603,012	5678	95.42
A3-2	11,872	98.0374	11,639	1288	39,786,654	6008	94.56
A3-3	11,872	96.6139	11,470	1035	40,166,166	6065	94.71
A1-1	11,872	85.3858	10,137	392	49,277,406	7441	94.78
B10-3	11,872	41.9643	4982	83	41,582,718	6279	95.19
A5-1	11,872	40.0775	4758	94	40,943,890	6183	94.85
B10-2	11,872	38.0475	4517	73	33,886,868	5117	94.98
B9-1	11,872	30.4835	3619	59	35,593,490	5375	95.07
B10-1	11,872	26.9794	3203	46	37,715,730	5695	95.36
A4-2	11,872	22.7257	2698	51	36,605,156	5527	94.69

One of the de novo-assembled contigs recovered from the individual library and sample containing 8,079 viral reads, was 11,811 nt in length and had 100% base coverage with the reference, thus representing a complete genome of the virus. It was 7 nt longer at the 5' end than ON669064, which was confirmed by 5' RACE using SMARTer® RACE 5'/3' Kit (Takara Bio USA, Inc., San Jose, CA). The 3’ end of the sequence was 59 nt longer than that of the reference ON669064 and matched another isolate of the virus, SRAV_ALF1071, found in GenBank under accession number ON669090.1 [1]. Application of the 3'RACE also showed that the virus has ~ 30-long 3′-terminal poly(A) tract, which is absent in all members of the family *Flaviviridae* ([14]; https://ictv.global/report/chapter/flaviviridae/flaviviridae).

At the nucleotide level, the SRAV-WA1 (for Washington State) was 99.8% identical to the reference genome ON669064 with 18 SNPs between the two, therefore depicting an isolate of the same virus. The genome of SRAV-WA1 encoded a single 3,835 amino acid (aa) polyprotein 99.9% identical to the reference. BLASTP and PSI-BLAST searchers of the SRAV-WA1 polyprotein against the GenBank database identified no related sequences except existing SRAV submissions. In silico analyses of the viral polyprotein using Pfam, InterPro and CDD databases revealed the presence of the conserved RdRp domain (3220–3478 aa, E-value = 1.43E-21, InterPro). No other domains were reliably identified. A weak relation to the superfamily of trypsin-like serine proteases (E-value = 1.01e-03) was found in the 1855–1910 aa region of the polyprotein when Superfamily database (https://supfam.org) was used to detect protein sequence similarities.

**Fig. 2:**
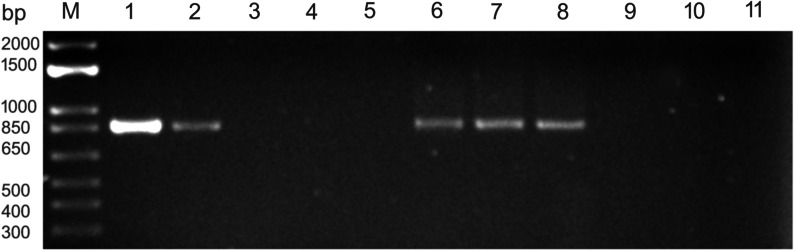
RT-PCR with primers specific for Snake River alfalfa virus. M, 1 kb plus DNA marker (Thermo Fisher Scientific, Waltham, MA). Lanes 1,2: RT-PCR products amplified with primers LN1036/37 and ANPV_3, respectively. Lanes 3,4 and 5: amplification from alfalfa samples containing no SRAV reads, water control, and Taq DNA polymerase (no RT mix added to verify the absence of genomic DNA), respectively; primers LN1036/37. Lanes 6, 7, and 8: representative RT-PCR products amplified from seeds of alfalfa cultivars SW-9215, CUF101, and Maverick using LN1036/37 primers. Lanes 9,10, and 11: the same reaction controls as shown in lanes 3–5

The results obtained by HTS were validated by RT-PCR with two sets of primers in three technical replicates using SuperScript III One-Step RT-PCR System (Thermo Fisher Scientific, Waltham, MA). One set was ANPV_3 derived from Dahan et al. [1], and another set of primers was designed in this work: LN1036-F, GGGAGAACCAGGAAACTGTTAG and LN1037-R, CTGTCGCATAGTCCGCTTATT. RT-PCR using both primers pairs produced correct amplicons, while no products were generated from control samples in which no SRAV-related HTS reads were found (Fig. 2). The amplicons were sequenced and validated to be SRAV.

The omnipresence of the virus in all analyzed samples indicated the possibility of persistent infection like those caused by partitiviruses and endornaviruses [16]. Considering resemblance in the size and structure of the genome, we compared SRAV to endornaviruses. Viruses in this family infect plants, fungi, and Oomycetes, and are generally associated with symptomless infections and no pathogenic effects [3]. They have a linear genome of 10 to 17 kbp in length, that encodes a polyprotein ranging from 3,217 to 5,825 aa [3, 16, 19]. Notably, several known endornaviruses, same as SRAV, lack helicase domain [16, 19]. While members of the *Endornaviridae* family were often reported as double-stranded RNA viruses [3, 16], current ICTV classification describes them as single-stranded, positive-sense RNA genomes that have been characterized using replicative dsRNAs forms [19].

Phylogenetic analysis using the polyproteins of SRAV, different viruses of the family *Endornaviridae*, and members of the family *Flaviviridae*, placed both SRAV isolates within *Endornaviridae*, although SRAV isolates formed a separate cluster (Additional File 1). When we performed phylogenetic analysis using InterPro-extracted RdRp domains of the *Endornaviridae* and *Flaviviridae* (3,204–3,462 aa in SRAV-WA1), SRAV clustered with the former as well, again forming a distinct grouping (Additional File 2). It is worth noting, however, that SRAV placement was not consistent, pointing to potentially incorrect phylogenies or an irreproducibility in maximum likelihood inference and [18].

When we used Sequence Demarcation Tool program that allows classification of virus sequences based on sequence pairwise identity (SDTv1.2; [10]), it showed low similarities of both polyprotein and RdRp of SRAV with those of endornaviruses and flaviviruses (Additional Files 3 and 4).

Since plant endornaviruses are transmitted through seeds via the gametes [8, 13, 19], we decided to test seeds of several alfalfa cultivars for the presence of the virus by RT-PCR. Seeds were scarified with concentrated H_2_SO_4_, surface-sterilized with 70% ethanol, and rinsed with sterile water [11]. Total RNA was extracted with Takara Plant and Fungal RNA isolation kit (Takara Bio, San Jose, CA) and used in RT-PCR with primers LN1036/37. RT-PCRs with five out of six tested seed samples derived from different alfalfa cultivars (Maverick, SW-9215, SW-8421, SW-9720, and CUF101) were virus-positive, indicating a high rate of seed infection (Fig. 2). Resultant amplicons were sequenced and confirmed to be SRAV-WA1. Seeds of one cultivar, Regency SY, were RT-PCR-negative (not shown). To additionally confirm seed transmission of the virus, leaves of the germinated seedlings were randomly checked by RT-PCR one week after germination. Except for Regency SY, seedlings of other tested cultivars were positive for SRAV-WA1 (not shown). These experiments demonstrated localization of SRAV-WA1 in the internal parts of the seed, likely in the embryo. They also showed a high rate of seed infection by the virus, and its efficient vertical transmission via seeds, thus confirming persistent nature of the virus [15].

**Fig. 3:**
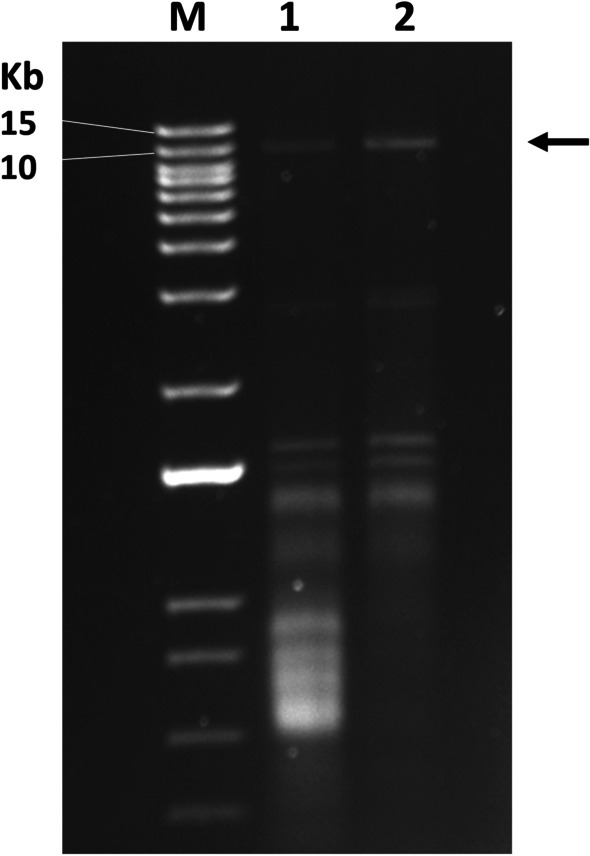
Agarose gel electrophoresis of dsRNAs extracted from leaves of two different alfalfa plants containing SRAV-WA1 reads. M, 1 kb plus DNA ladder (Thermo Fisher Scientific, Waltham, MA). Lane 1, dsRNA extracted from the sample A2-2 (Table 1). Lane 2, dsRNA extracted from the sample B8-2 (Table 1). Arrow indicates the predicted dsRNA of SRAV-WA1

One of the characteristic features of all endornaviruses is readily detectable viral replicative form, double-stranded RNAs (dsRNAs), that accumulates in the host tissues in high quantities [19]. To extract dsRNA from leaves of the SRAV-WA1-infected alfalfa plants, we followed the protocol of Khankhum et al. [5]. Agarose gel electrophoresis showed the presence of dsRNA of the approximately correct size corresponding to that predicted by de novo assembly of the HTS reads (Fig. 3).

Additionally, given that many endornavirus RNAs have a site-specific discontinuity (nick) on 5’ terminus of the coding strand, we have attempted, but failed, to determine its presence in the genome of SRAV-WA1 using 5'RACE approach [13].

Dahan et al. [1] detected SRAV in western flower thrips and suggested a possible role for the insect in virus transmission. However, thrips are known to transmit tospoviruses and plant viruses in the *Ilarvirus, Carmovirus, Sobemovirus* and *Machlomovirus* genera [4]. Based on our data, SRAV, analogously to vertically transmitted endornaviruses [8, 9, 13, 19], is also transmitted by seeds. Although alfalfa is one of the primary hosts for western flower thrips (and other species) and acquiring the SRAV during feeding cannot be excluded, transmission of the virus by thrips would require additional experimental confirmation.

Other viruses found in samples infected with SRAV-WA1 included alfalfa mosaic virus, pea streak virus, lucerne transient streak virus, bean leaf roll virus, partitiviruses, and amalgavirus. Fungal and bacterial pathogens, described in alfalfa, like *Alternaria* spp., *Bipolaris* spp., *Stemphylium* spp., *Fusarium* spp., *Pseudomonas* spp., *Erwinia* spp. etc. were also detected. These findings suggested that traditional Koch’s postulate of “one microbe—one disease” should be broadened into the principle of a pathobiome, when disease symptoms are attributed to a diverse community of pathogenic organisms within the plant, rather than to a single infectious agent [20].

Overall, our research confirmed association of SRAV with alfalfa and, for the first time, identified an extensive occurrence of this virus in Washington State. The importance of this work also relies on the hypothesis that placement of SRAV within the flavi-like lineage, as suggested by Dahan et al. [1], may not be entirely accurate. Prevalence of the virus in alfalfa plants, its genome organization, seed-mediated transmission, presence of the easily detectable dsRNA and, although partly, phylogenetic reconstruction, suggest that SRAV is a persistent virus possessing some features characteristic for endornaviruses.

However, the low percent identity of SRAV with endornaviruses and flaviviruses, absence of the poly (C) and presence of the poly (A) tract at the 3’ terminus of the genome, and lack of the site-specific nick at the 5’ end, indicate that SRAV may represent an entirely new taxonomic group of persistent viruses that does not belong to either of the two families. Altogether, more data are needed to assess taxonomy, biology, and economic importance of the virus.

## Supplementary Information

**Additional file 1**: Phylogenetic relationship of SRAV with members of the families *Endornaviridae* and *Flaviviridae*. The original unrooted tree was deduced from MUSCLE alignment [2] of the viral polyproteins and built using MEGA X software with Maximum Likelihood method and bootstrap analysis of 1000 replicates.

**Additional file 2**: Phylogenetic relationship of SRAV with members of the families *Endornaviridae* and *Flaviviridae*. The unrooted tree was deduced from MUSCLE alignment of the viral RdRP domains and built using MEGA software with Maximum Likelihood method and bootstrap analysis of 1000 replicates.

**Additional file 3**: Color coded matrix of pairwise similarity scores obtained with Sequence Demarcation Tool Version 1.2 (SDTv1.2). Polyproteins of the representative endorna- and flaviviruses were aligned using MUSCLE program [2].

**Additional file 4**: Color coded matrix of pairwise similarity scores obtained with Sequence Demarcation Tool Version 1.2 (SDTv1.2). RdRp domains of the representative endorna- and flaviviruses were aligned using MUSCLE program [2].

## Data Availability

The complete genomic sequence of the SRAV (SRAV-WA1 isolate) has been deposited in GenBank under the accession number OP321578.

## Abbreviations

SRAV: Snake River alfalfa virus
RdRp: RNA-dependent RNA polymerase
HTS: High-throughput sequencing
HMM: Hidden Markov Models
RACE: Rapid Amplification of cDNA Ends
RT-PCR: Reverse transcription-polymerase chain reaction

## References

[1] Dahan J, Wolf YI, Orellana GE, Wenninger EJ, Koonin EV, Karasev AV (2022). A novel flavi-like virus in Alfalfa (*Medicago sativa* L.) crops along the Snake River Valley. Viruses.

[2] Edgar RC (2004). MUSCLE: multiple sequence alignment with high accuracy and high throughput. Nucleic Acids Res.

[3] Fukuhara T (2019). Endornaviruses: persistent dsRNA viruses with symbiotic properties in diverse eukaryotes. Virus Genes.

[4] Jones DR (2005). Plant viruses transmitted by thrips. Eur J Plant Pathol.

[5] Khankhum S, Escalante C, de Rodrigues SE, Valverde RA (2017). Extraction and electrophoretic analysis of large dsRNAs from desiccated plant tissues infected with plant viruses and biotrophic fungi. Eur J Plant Pathol.

[6] Kumar S, Stecher G, Li M, Knyaz C, Tamura K (2018). MEGA X: molecular evolutionary genetics analysis across computing platforms. Mol Biol Evol.

[7] Meleshko D, Mohimani H, Tracanna V, Hajirasouliha I, Medema MH, Korobeynikov A, Pevzner PA (2019). BiosyntheticSPAdes: reconstructing biosynthetic gene clusters from assembly graphs. Genome Res.

[8] Moriyama H, Kanaya K, Wang JZ, Nitta T, Fukuhara T (1996). Stringently and developmentally regulated levels of a cytoplasmic double-stranded RNA and its high-efficiency transmission via egg and pollen in rice. Plant Mol Biol.

[9] Moriyama H, Horiuchi H, Nitta T, Fukuhara T (1999). Unusual inheritance of evolutionarily-related double-stranded RNAs in interspecific hybrid between rice plants Oryza sativa and Oryza rufipogon. Plant Mol Biol.

[10] Muhire BM, Varsani A, Martin DP (2014). SDT: a virus classification tool based on pairwise sequence alignment and identity calculation. PLoS ONE..

[11] Nemchinov LG, Grinstead S (2020). Identification of a novel isolate of Alfalfa virus S from China suggests a possible role of seed contamination in the distribution of the virus. Plant Dis.

[12] Okada R, Kiyota E, Sabanadzovic S, Moriyama H, Fukuhara T, Saha P, Roossinck MJ, Severin A, Valverde RA (2011). Bell pepper endornavirus: molecular and biological properties, and occurrence in the genus Capsicum. J Gen Virol.

[13] Okada R, Yong CK, Valverde RA, Sabanadzovic S, Aoki N, Hotate S, Kiyota E, Moriyama H, Fukuhara T (2013). Molecular characterization of two evolutionarily distinct endornaviruses co-infecting common bean (*Phaseolus vulgaris*). J Gen Virol.

[14] Payne S (2017). Family *Flaviviridae*. Viruses, from understanding to investigation.

[15] Roossinck MJ (2010). Lifestyles of plant viruses. Philos Trans R Soc Lond B Biol Sci.

[16] Roossinck MJ, Sabanadzovic S, Okada R, Valverde RA (2011). The remarkable evolutionary history of endornaviruses. J Gen Virol.

[17] Roossinck MJ (2015). A new look at plant viruses and their potential beneficial rolesin crops. Mol Plant Pathol.

[18] Shen XX, Li Y, Hittinger CT, Chen XX, Rokas A (2020). An investigation of irreproducibility in maximum likelihood phylogenetic inference. Nature Comm.

[19] Valverde RA, Khalifa ME, Okada R, Fukuhara T, Sabanadzovic S (2019). ICTV virus taxonomy profile: endornaviridae. J Gen Virol.

[20] Vayssier-Taussat M, Albina E, Citti C, Cosson JF, Jacques MA, Lebrun MH, Le Loir Y, Ogliastro M, Petit MA, Roumagnac P, Candresse T (2014). Shifting the paradigm from pathogens to pathobiome: new concepts in the light of meta-omics. Front Cell Infect Microbiol.

