# Supplementary figures and images for: Snake River alfalfa virus, a persistent virus infecting alfalfa (Medicago sativa L.) in Washington State, USA
Source: Virol J. 2023 Feb 19;20:32. doi: 10.1186/s12985-023-01991-7 (PMC9938972; doi:10.1186/s12985-023-01991-7)

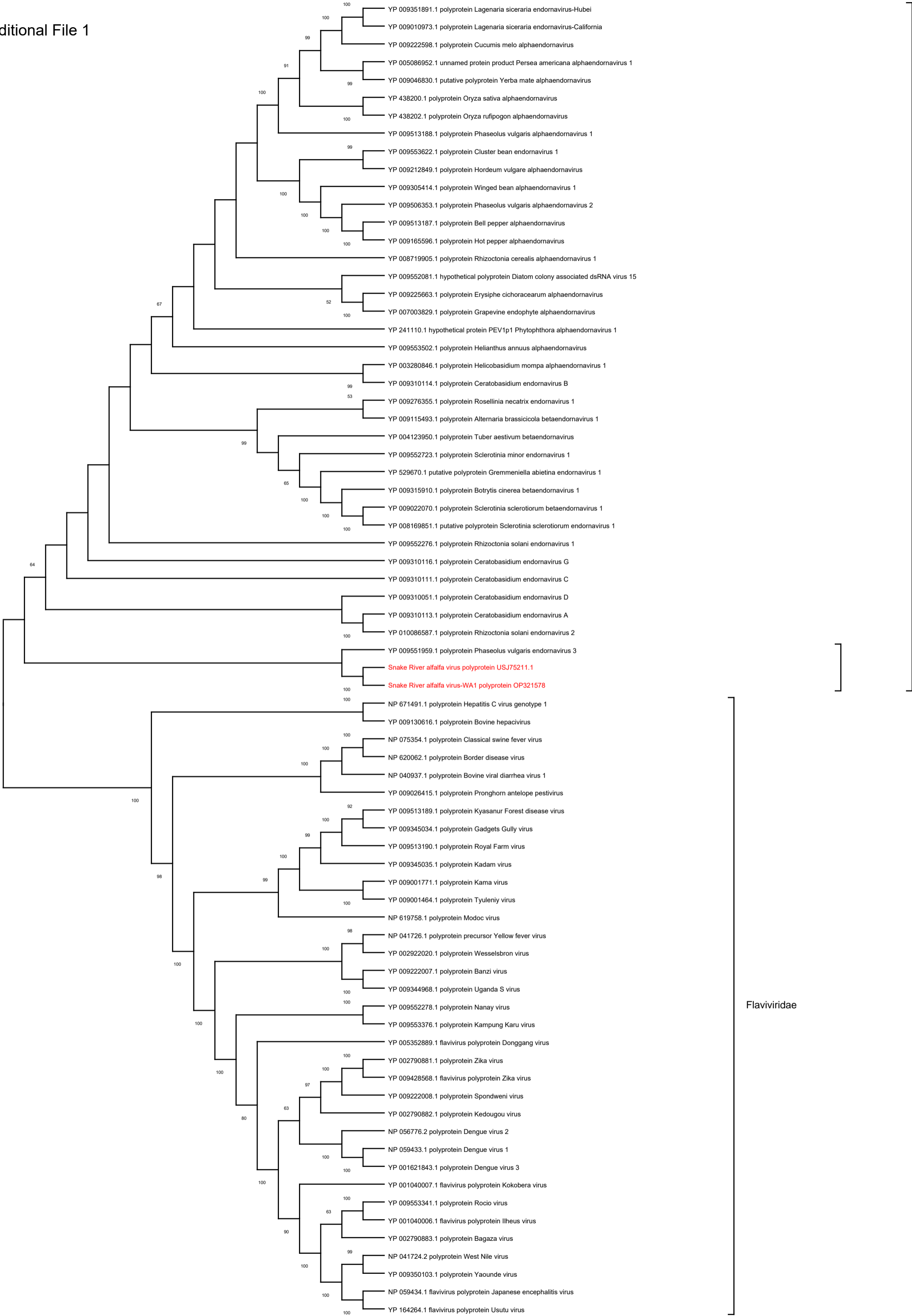

Supplement: Supplementary file 1 — Additional file 1: Phylogenetic relationship of SRAV with members of the families Endornaviridae and Flaviviridae. The original unrooted tree was deduced from MUSCLE alignment [2] of the viral polyproteins and built using MEGA X software with Maximum Likelihood method and bootstrap analysis of 1000 replicates. [file 12985_2023_1991_MOESM1_ESM.pdf]

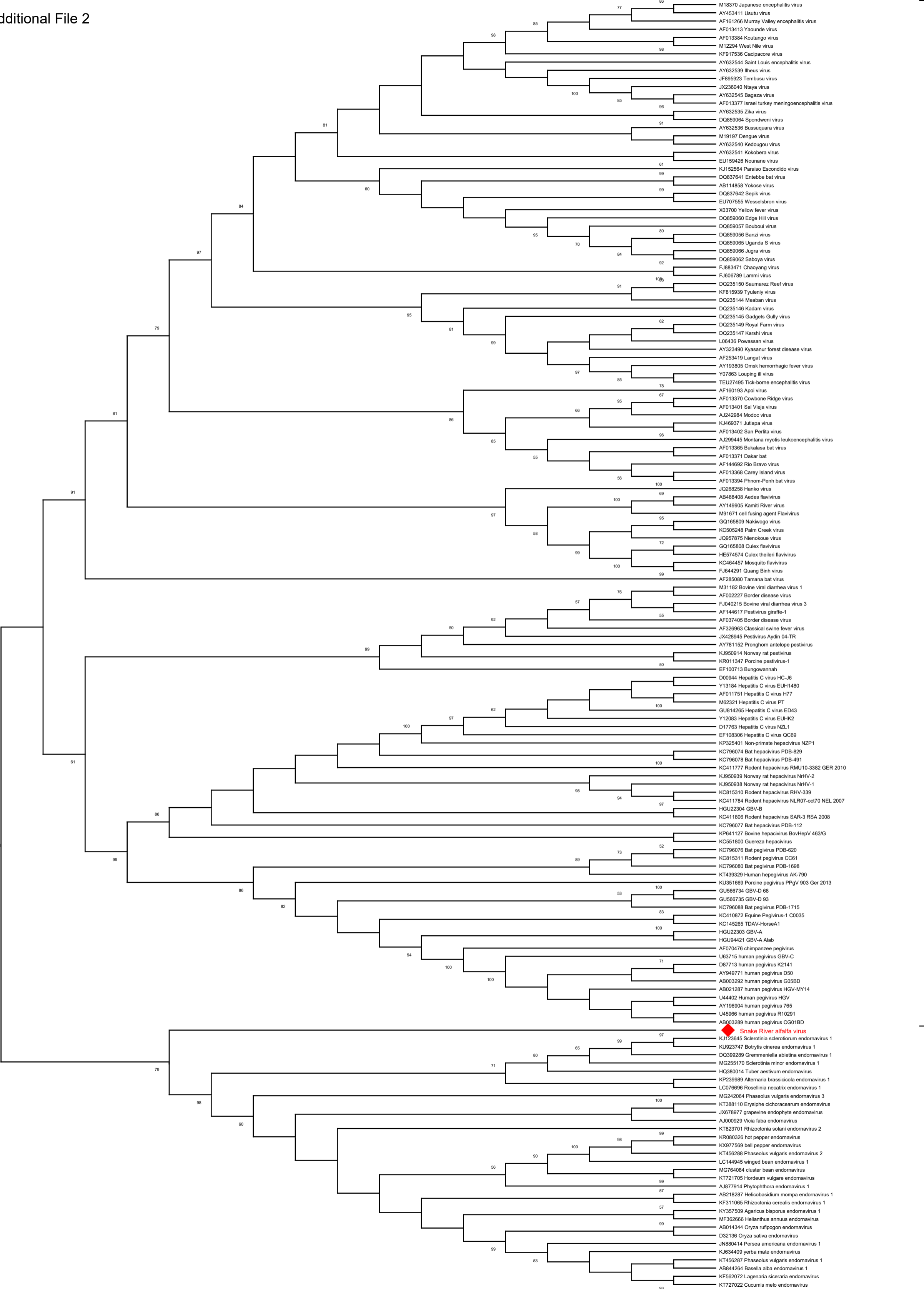

Flaviviridae

Endornaviridae

Supplement: Supplementary file 2 — Additional file 2: Phylogenetic relationship of SRAV with members of the families Endornaviridae and Flaviviridae. The unrooted tree was deduced from MUSCLE alignment of the viral RdRP domains and built using MEGA software with Maximum Likelihood method and bootstrap analysis of 1000 replicates. [file 12985_2023_1991_MOESM2_ESM.pdf]
