# Supplementary material for: Snake River alfalfa virus, a persistent virus infecting alfalfa (Medicago sativa L.) in Washington State, USA
Source: Virol J. 2023 Feb 19;20:32. doi: 10.1186/s12985-023-01991-7 (PMC9938972; doi:10.1186/s12985-023-01991-7)

Additional File 4

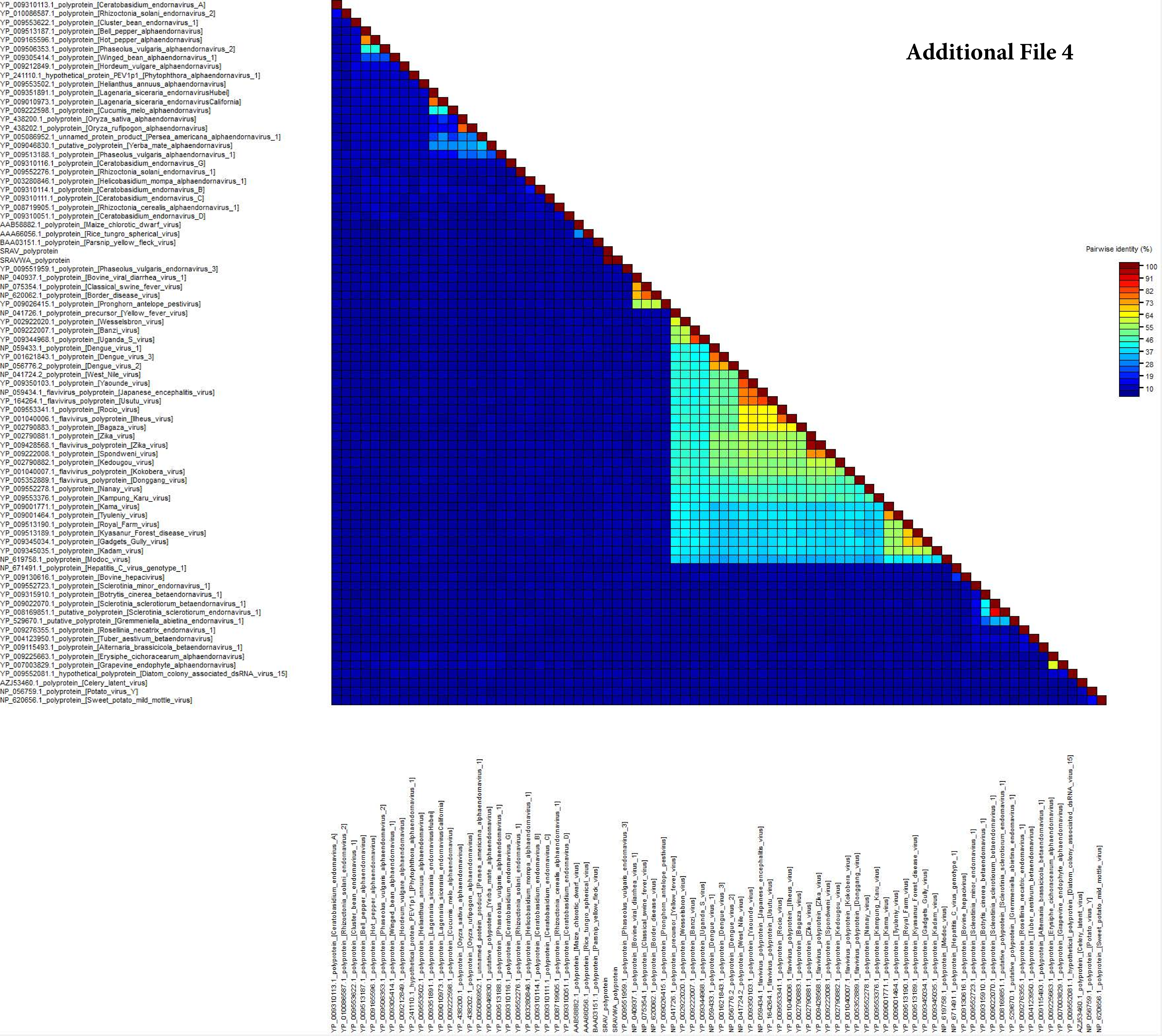

Supplement: Supplementary file 3 — Additional file 3: Color coded matrix of pairwise similarity scores obtained with Sequence Demarcation Tool Version 1.2 (SDTv1.2). Polyproteins of the representative endorna- and flaviviruses were aligned using MUSCLE program [2]. [file 12985_2023_1991_MOESM3_ESM.pdf]
