# Supplementary material for: Snake River alfalfa virus, a persistent virus infecting alfalfa (Medicago sativa L.) in Washington State, USA
Source: Virol J. 2023 Feb 19;20:32. doi: 10.1186/s12985-023-01991-7 (PMC9938972; doi:10.1186/s12985-023-01991-7)
Supplement: Supplementary file 4 — Additional file 4: Color coded matrix of pairwise similarity scores obtained with Sequence Demarcation Tool Version 1.2 (SDTv1.2). RdRp domains of the representative endorna- and flaviviruses were aligned using MUSCLE program [2]. [file 12985_2023_1991_MOESM4_ESM.pdf]

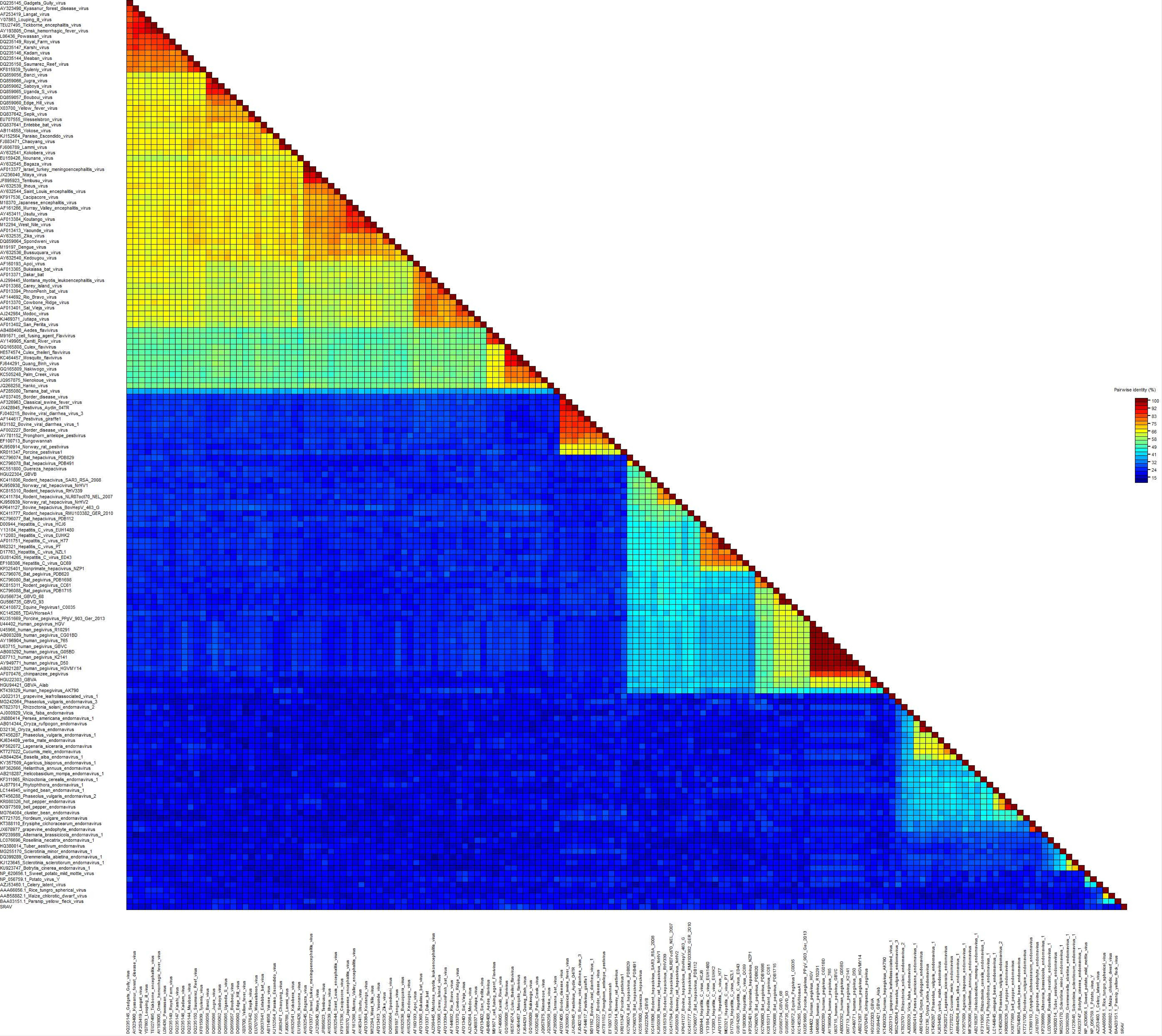
Pairwise identity (%)
100
92
83
75
66
58
49
41
32
24
15
DQ235145_Gadgets_Gully_virus
AY323490_Kyasanur_forest_disease_virus
AF253419_Langat_virus
Y07863_Louping_ill_virus
TEU27495_Tickborne_encephalitis_virus
AY193805_Omsk_hemorrhagic_fever_virus
L06436_Powassan_virus
DQ235149_Royal_Farm_virus
DQ235147_Karshi_virus
DQ235146_Kadam_virus
DQ235144_Meaban_virus
DQ235150_Saumarez_Reef_virus
KF815939_Tyuleniy_virus
DQ859056_Banzi_virus
DQ859066_Jugra_virus
DQ859062_Saboya_virus
DQ859065_Uganda_S_virus
DQ859057_Bouboui_virus
DQ859060_Edge_Hill_virus
X03700_Yellow_fever_virus
DQ837642_Sepik_virus
EU707555_Wesselsbron_virus
DQ837641_Entebbe_bat_virus
AB114858_Yokose_virus
KJ152564_Paraiso_Escondido_virus
FJ883471_Chaoyang_virus
FJ606789_Lammi_virus
AY632541_Kokobera_virus
EU159426_Nounane_virus
AY632545_Bagaza_virus
AF013377_Israel_turkey_meningoencephalitis_virus
JX236040_Ntaya_virus
JF895923_Tembusu_virus
AY632539_Ilheus_virus
AY632544_Saint_Louis_encephalitis_virus
KF917536_Cacipacore_virus
M18370_Japanese_encephalitis_virus
AF161266_Murray_Valley_encephalitis_virus
AY453411_Usutu_virus
AF013384_Koutango_virus
M12294_West_Nile_virus
AF013413_Yaounde_virus
AY632535_Zika_virus
DQ859064_Spondweni_virus
M19197_Dengue_virus
AY632536_Bussuquara_virus
AY632540_Kedougou_virus
AF160193_Apoi_virus
AF013365_Bukalasa_bat_virus
AF013371_Dakar_bat
AJ299445_Montana_myotis_leukoencephalitis_virus
AF013368_Carey_Island_virus
AF013394_PhnomPenh_bat_virus
AF144692_Rio_Bravo_virus
AF013370_Cowbone_Ridge_virus
AF013401_Sal_Vieja_virus
AJ242984_Modoc_virus
KJ469371_Jutiapa_virus
AF013402_San_Perlita_virus
AB488408_Aedes_flavivirus
M91671_cell_fusing_agent_Flavivirus
AY149905_Kamiti_River_virus
GQ165808_Culex_flavivirus
HE574574_Culex_theileri_flavivirus
KC464457_Mosquito_flavivirus
FJ644291_Quang_Binh_virus
GQ165809_Nakiwogo_virus
KC505248_Palm_Creek_virus
JQ957875_Nienokoue_virus
JQ268258_Hanko_virus
AF285080_Tamana_bat_virus
AF037405_Border_disease_virus
AF326963_Classical_swine_fever_virus
JX428945_Pestivirus_Aydin_04TR
FJ040215_Bovine_viral_diarrhea_virus_3
AF144617_Pestivirus_giraffe1
M31182_Bovine_viral_diarrhea_virus_1
AF002227_Border_disease_virus
AY781152_Pronghorn_antelope_pestivirus
EF100713_Bungowannah
KJ950914_Norway_rat_pestivirus
KR011347_Porcine_pestivirus1
KC796074_Bat_hepacivirus_PDB829
KC796078_Bat_hepacivirus_PDB491
KC551800_Guereza_hepacivirus
HGU22304_GBVB
KC411806_Rodent_hepacivirus_SAR3_RSA_2008
KJ950938_Norway_rat_hepacivirus_NrHV1
KC815310_Rodent_hepacivirus_RHV339
KC411784_Rodent_hepacivirus_NLR07oct70_NEL_2007
KJ950939_Norway_rat_hepacivirus_NrHV2
KP641127_Bovine_hepacivirus_BovHepV_463_G
KC411777_Rodent_hepacivirus_RMU103382_GER_2010
KC796077_Bat_hepacivirus_PDB112
D00944_Hepatitis_C_virus_HCJ6
Y13184_Hepatitis_C_virus_EUH1480
Y12083_Hepatitis_C_virus_EUHK2
AF011751_Hepatitis_C_virus_H77
M62321_Hepatitis_C_virus_PT
D17763_Hepatitis_C_virus_NZL1
GU814265_Hepatitis_C_virus_ED43
EF108306_Hepatitis_C_virus_QC69
KP325401_Nonprimate_hepacivirus_NZP1
KC796076_Bat_pegivirus_PDB620
KC796080_Bat_pegivirus_PDB1698
KC815311_Rodent_pegivirus_CC61
KC796088_Bat_pegivirus_PDB1715
GU566734_GBVD_68
GU566735_GBVD_93
KC410872_Equine_Pegivirus1_C0035
KC145265_TDAVHorseA1
KU351669_Porcine_pegivirus_PPgV_903_Ger_2013
U44402_Human_pegivirus_HGV
U45966_human_pegivirus_R10291
AB003289_human_pegivirus_CG01BD
AY196904_human_pegivirus_765
U63715_human_pegivirus_GBVC
AB003292_human_pegivirus_G05BD
D87713_human_pegivirus_K2141
AY949771_human_pegivirus_D50
AB021287_human_pegivirus_HGVMY14
AF070476_chimpanzee_pegivirus
HGU22303_GBVA
HGU94421_GBVA_Alab
KT439329_Human_hepegivirus_AK790
JQ023131_grapevine_leafrollassociated_virus_1
MG242064_Phaseolus_vulgaris_endornavirus_3
KT823701_Rhizoctonia_solani_endornavirus_2
AJ000929_Vicia_faba_endornavirus
JN880414_Persea_americana_endornavirus_1
AB014344_Oryza_rufipogon_endornavirus
D32136_Oryza_sativa_endornavirus
KT456287_Phaseolus_vulgaris_endornavirus_1
KJ634409_yerba_mate_endornavirus
KF562072_Lagenaria_siceraria_endornavirus
KT727022_Cucumis_melo_endornavirus
AB844264_Basella_alba_endornavirus_1
KY357509_Agaricus_bisporus_endornavirus_1
MF362666_Helianthus_annuus_endornavirus
AB218287_Helicobasidium_mompa_endornavirus_1
KF311065_Rhizoctonia_cerealis_endornavirus_1
AJ877914_Phytophthora_endornavirus_1
LC144945_winged_bean_endornavirus_1
KT456288_Phaseolus_vulgaris_endornavirus_2
KR080326_hot_pepper_endornavirus
KX977569_bell_pepper_endornavirus
MG764084_cluster_bean_endornavirus
KT721705_Hordeum_vulgare_endornavirus
KT388110_Erysiphe_cichoracearum_endornavirus
JX678977_grapevine_endophyte_endornavirus
KP239989_Alternaria_brassicicola_endornavirus_1
LC076696_Rosellinia_necatrix_endornavirus_1
HQ380014_Tuber_aestivum_endornavirus
MG255170_Sclerotinia_minor_endornavirus_1
DQ399289_Gremmeniella_abietina_endornavirus_1
KJ123645_Sclerotinia_sclerotiorum_endornavirus_1
KU923747_Botrytis_cinerea_endornavirus_1
NP_620656.1_Sweet_potato_mild_mottle_virus
NP_056759.1_Potato_virus_Y
AZJ53460.1_Celery_latent_virus
AAA66056.1_Rice_tungro_spherical_virus
AAB58882.1_Maize_chlorotic_dwarf_virus
BAA03151.1_Parsnip_yellow_fleck_virus
SRAV
